# On the Mortality and Mean Length of Abode of the Patients in the Large Hospital of Milan from 1811 to 1844, and in That of the Brothers of Charity from 1604 to 1844, &c.

**Published:** 1847-10

**Authors:** 


### Art. VII.

*Della Mortalità e dimora media dei malati nello Spedale Maggiore di Milana dal* 1811 *al* 1844, *ed in quello dei RR. Fatebenefratelli dal* 1604 *al* 1844, *coi prospetti del calcolo compelessivo sopra* 784,000 *infermi.* Memoria del Medico-Statista Dottore Guiseppe Ferrario, di Milano.—*Milano,* 1845.

*On the Mortality and Mean Length of Abode of the Patients in the Large Hospital of Milan from* 1811 *to* 1844, *and in that of the Brothers of Charity from* 1604 *to* 1844, *&c.* A Memoir, by Dr. G. Ferrario, Medical Statist, of Milan.—*Milan,* 1845. 8vo, pp. 16.

This pamphlet was prepared for communication to the medical section of the sixth congress of the Italian Association of Science, held in Milan in September, 1844, and the author has published it with the hope that others will be induced to follow his example, and that thus a vast series of facts may be obtained, upon which the hospitals of different regions may be compared. He thinks at least, that great practical utility must result from the establishment of various *laws of probability,* according to which males and females suffering from ordinary diseases, die or are cured monthly, and annually in different places. He would wish to establish in every place where an hospital or dispensary exists, a long and continued series of exact statistical annotations, showing the ordinary mortality and proportion of cures upon every 100 sick, monthly and annually. He thinks that from this practical corollaries might be deduced of great value, and then rational experiments might be made to determine the value of different modes of treatment.

The following conclusions are drawn from his first table of the mortality, &c. in the large hospital at Milan, during thirty years.

1. The mortality of the sick in the great hospital of Milan, monthly and annually, is constantly greater in females than in males, in the proportion of 2 to 6 per cent.

2. The mortality from November to the end of April (that is, the whole winter and spring) varies in males from 14 to 19 per cent.; in females from 16½ to 24½ per cent.; and the total mortality of the two sexes from 15 to 21 per cent.

3. The mortality from May to the end of October, (that is, summer and autumn), in males, varies from 7½ to 12 per cent.; in females, from 11½ to 14½; and in both together, from 9½ to 13 per cent.

4. During the whole period, the mortality of males, and of both sexes taken together, is at the minimum in September, it begins to increase in October, and continues to increase until it attains its maximum in January. It begins to decrease in February, and continues to do so in the succeeding months of spring and summer, and attains its minimum in August, and particularly in September.

5. The mortality of females calculated alone in the six months of winter and spring, proceeds in exact proportion with that of the males, but differs in the other months. The minimum mortality of females is in August, and begins to increase in September, proving that females suffer more quickly than males from the morbid influences of autumn, and from the cold of winter.

6. The mean annual mortality of males was 12·41; and of females, 16·13; and of both sexes, 13·82 per cent., including those brought dead, or in the last moments of life to the hospital. It is to be observed, that about 400 chronic incurables are always in this hospital.

We need not follow the accounts from the other hospital, but it appears that the facts just stated exactly agree with those deduced from the second table.

The stay in hospital was always longer in females, exceeding at least two days that of males; and in August four days. The mean stay during the thirty years, was 13 days for males and 15 for females.

It is stated that the Milanese females are much less willing than the males to go to hospital, preferring rather to be treated at home by the permanent physicians for the poor. Their number in hospital is always less than that of males, and their diseases are in a more advanced stage.

In the other hospital the mean stay was 21½ days. This increased time is owing to the convalescents being detained for a longer period, as there is a large ward set apart for them, and they are selected from the better class of the lower orders, poor but not destitute, whereas no distinction of this kind is observed in the larger establishment.

Dr. Ferrario is inclined to believe that the fact of such a mass of patients being discharged about the 14th and 21st day, supports the hippocratic doctrine of critical days. It must be remembered that patients are not brought to hospital until some days after the commencement of disease; but this is compensated for by the days of convalescence, and thus the mean stay of 14 days in one hospital, and 21 in the other, is supposed to be in relation with critical days on the seventh. But in our opinion the facts by no means warrant any such inference. Of 400 chronic incurables constantly in hospital, and who leave it only when death frees them from their sufferings, it must be quite clear that a large number must considerably exceed a stay of three weeks, and may probably remain several months. Now to bring the general average down to 14 or 21 days, it is also evident that a considerable number must be discharged within the lowest of those periods, and thus it appears very doubtful if the mean number would represent the actual stay in hospital of the majority of patients. If he had wished to examine this question, the author should have discovered the number of patients discharged from the 1st to the 7th day; the 7th to the 14th; the 14th to the 21st, and so on; and if any considerable majority were discharged on any particular day in either of these periods. This inquiry might be also advantageously employed comparatively in fever hospitals, and those in which other diseases are treated.

If patients are discharged on a particular day of the week in Tuscany as they are in England, this may account for the terms of residence being multiples of seven.

The author is of opinion that great benefit would result from trials of any given remedy, or plan of treatment, in the same months in all the principal cities of Europe; and that thus some appreciation might be made of meteorological influences in modifying the effect of remedies, by comparing the mean mortality and duration of treatment. But there are many and obvious difficulties and impediments to the execution of any such plan. There might arise questions as to accuracy of diagnosis, previous condition of patient, and the endless modifications of diseases quite beyond the reach of the statistician, and referrible to many other moral and physical causes, totally independent of climate or weather. The inquiry is nevertheless quite as well worthy of simultaneous notice as the meteorological observations, suggested by Sir John Herschel. The relative success of various surgical operations in different localities, and at different seasons, should also be investigated. Something must evidently be done to relieve medicine from the uncertainties under which it labours; and we know of no other way in which this can be accomplished, than by making extensive observations on a uniform plan, of such facts as admit of exact determination and statement.

We need not say more with regard to the pamphlet before us, than that the tables appear to be prepared with much care and industry.

---

